# Malignant Hemangiopericytoma Arising in Neurofibromatosis: A Case Report with Histological, Immunohistochemical and Ultrastructural Studies

**DOI:** 10.1080/13577149977776

**Published:** 1999-09

**Authors:** Jun Wang, Hernan Vargas, Karl Gaal, Xia Wang, Shi-Kaung Peng

**Affiliations:** 1 Department of Pathology Harbor-UCLA Medical Center University of California Los Angeles, School of Medicine 1000W. Carson Street Torrance CA 90509 USA; 2 Department of Surgery Harbor-UCLA Medical Center University of California Los Angeles, School of Medicine 1000W. Carson Street Torrance CA 90509 USA

## Abstract

*Subject.* A 27-year-old Hispanic male with clinical manifestation of neurofibromatosis type 1 developed chronic constipation and urination difficulty along with recently increased abdominal bloating and anorexia. He also noted 40 lbs weight loss over period of 1 year. Physical and radiographic examinations revealed a large mass in the right pelvic fossa.

*Results.* The surgically removed tumor was demonstrated, histologically, immunohistochemically, and ultrastructurally, to be a malignant hemangiopericytoma.

*Discussion.*Although non-neurogenic tumors associated with neurofibromatosis have been reported in these patients, only one hemangiopericytoma case has been found in the English literature. We report here another case of this rare malignant hemangiopericytoma in a patient with neurofibromatosis.


*Sarcoma* (1999) **3**, 135–139

CASE REPORT

### Malignant hemangiopericytoma arising in neurofibromatosis: a case report with histological, immunohistochemical and ultrastructural studies

JUN WANG,[1] HERNAN VARGAS,[2] KARL GAAL,[1] XIA WANG[1] & SHI-KAUNG PENG[1]

*Departments of [1]Pathology and [2]Surgery, Harbor-UCLA Medical Center, University of California Los Angeles, School of Medicine, 1000 W. Carson Street, Torrance, CA 90509, USA*

**Abstract**

*Subject.* A 27-year-old Hispanic male with clinical manifestation of neurofibromatosis type 1 developed chronic constipation and urination difficulty along with recently increased abdominal bloating and anorexia. He also noted 40 lbs weight loss over period of 1 year. Physical and radiographic examinations revealed a large mass in the right pelvic fossa.

*Results.* The surgically removed tumor was demonstrated, histologically, immunohistochemically, and ultrastructurally, to be a malignant hemangiopericytoma.

*Discussion.* Although non-neurogenic tumors associated with neurofibromatosis have been reported in these patients, only one hemangiopericytoma case has been found in the English literature. We report here another case of this rare malignant hemangiopericytoma in a patient with neurofibromatosis.

**Key words**: *hemangiopericytoma, neurofibromatosis*

### Introduction

Neurofibromatosis (NF), or von Recklinghausen's disease, is an autosomal dominant disorder characterized by multiple cafe-au-lait spots, cutaneous neurofibromas, and skeletal abnormalities, which may also be associated with various neurogenic malignancies.[1] Although rare, other non-neurogenic sarcomas, such as osteosarcoma, chondrosarcoma, liposarcoma, rhabdomyosarcoma, and angiosarcoma, have been reported in association with NF.[2–4] In a search of the English literature, we could only find one case of a solitary hemangiopericytoma arising in the ileum of a patient with von Recklinghausen's disease.[5] Hemangiopericytoma is an uncommon tumor derived from vascular pericytes and usually presents as a deep-seated mass in the retroperitoneum, pelvic fossa, lower limbs, orbital or sinonasal regions, or rarely the deep subcutis.[6] We report here a case of malignant hemangiopericytoma arising in pelvic fossa of a patient with NF-1. The histological, immunohistochemical, and ultrastructural features of hemangiopericytoma, as well as its differential diagnosis from other tumors with hemangiopericytoma-like pattern arising in patients with NF-1, are also reviewed.

### Patient

### *Clinical history*

A 27-year-old Hispanic male with NF-1 since birth presented with a 6-month history of chronic constipation and urination difficulty along with recently increased abdominal bloating and anorexia. He also noted 40 lbs weight loss over period of 1 year, occasional nausea and vomiting, and occasional hematochezia. He denied any hematemesis. His past medical history was significant for neurofibromatosis, type 1. The past surgical history included excision of right leg neurofibroma 1 month previously, sciatic nerve schwannoma 1 year previously, and biopsied neurofibroma of the right eyebrow 10 years previously. Family history revealed that mother and uncle had cafe-au-lait spots but without neurofibromatosis.

Physical examination revealed a thin Hispanic male with multiple neurofibromatous nodules on scalp, trunk, and extremities. Cafe-au-lait spots were observed on the right neck area but no Lisch spots were found. A large palpable right pelvic mass was found displacing the rectum anteriorly and medially on abdominal and rectal examination. The Guaiac test was positive. Right foot drop was noted.

Correspondence to: Shi-Kaung Peng, Department of Pathology, Harbor-UCLA Medical Center, 1000 W. Carson Street, Torrance, CA 90509, USA. Tel: +1 310 222 2205; Fax: +1 310 618 0249.

1357-714X print/1369-1643 online/99/020135-05 © 1999 Taylor & Francis Ltd

Computed tomography (CT) and magnetic resonance imaging (MRI) revealed a large mass in the right pelvic fossa (Fig. 1A). The patient underwent exploratory laparotomy and was found to have a large solitary tumor in the right pelvic fossa, which involved the internal iliac vessels and compressed the sacral plexus. The tumor was separated from the sacral nerves and removed surgically. The neurological deficit of the right foot improved slightly after surgery.

*Material, methods and results*

The tumor was sectioned extensively and the specimen was processed according to a standard histological method. Immunohistochemical staining was performed using a universal biotin–streptavidin peroxidase system (Vector Laboratories, Inc., Burlingame) with commercially available antibodies. The antibodies used were against cytokeratin CAM5.2 (Becton-Dickinson, San Jose, CA) and AE1/AE3

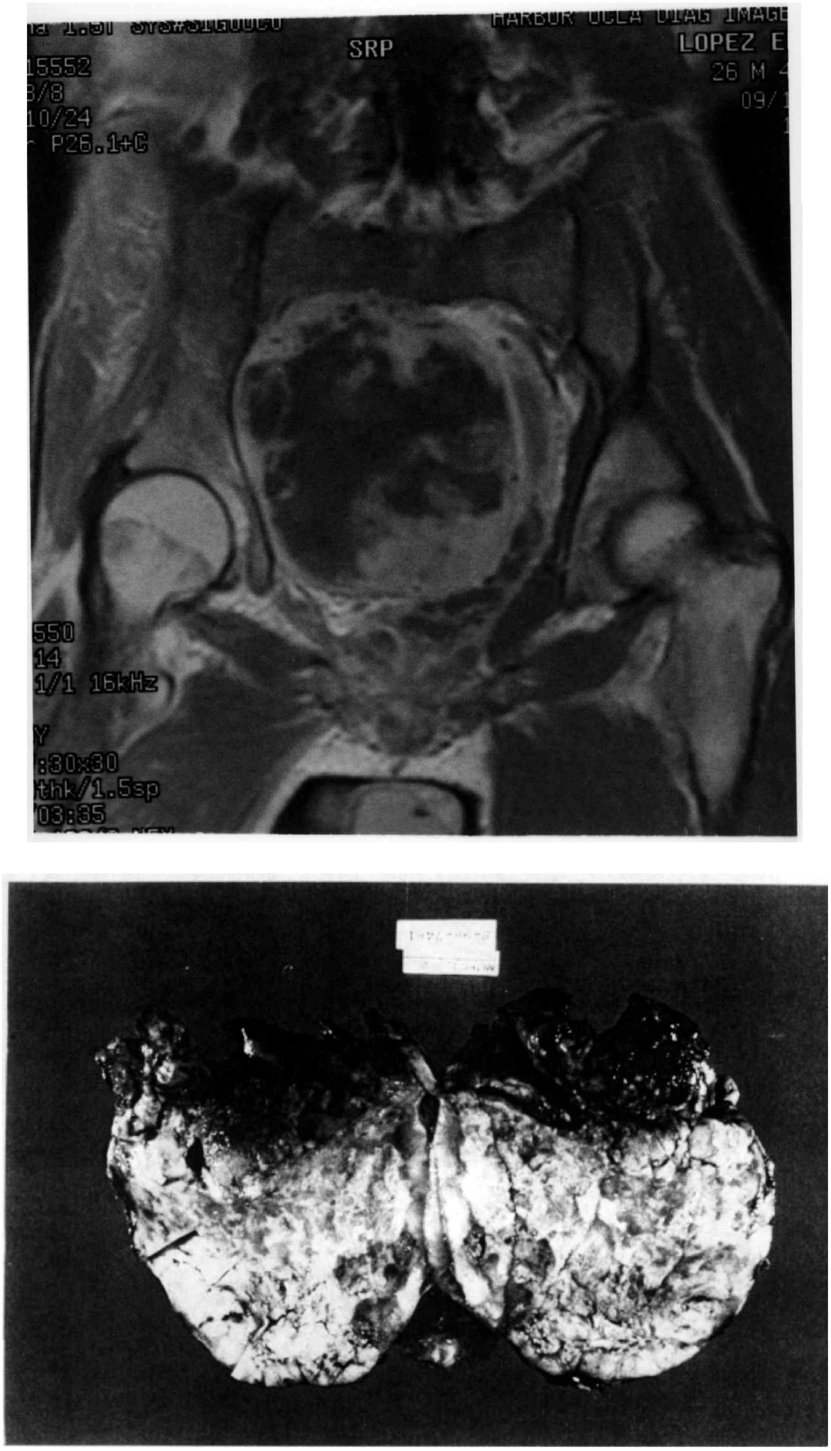

**Fig. 1.** *(A) Magnetic resonance imaging showed a large pelvic mass displacing rectum medially and anteriorly. (B) Bisected tumor mass revealed a variegated tan light yellow to tan brown cut surface with extensive necrosis, focal hemorrhage and gelatinous degeneration.*

(Boehringer Mannheim Corporation, Indianapolis, IN), α-actin (Sigma, St. Louis, MO), actin (Enzo Diagnostic, Inc., Syosset, NY), desmin (Dako), myoglobin (Dako), collagen IV (Dako), factor XIIIa (Calbiochem, San Diego, CA), vimentin (Sigma), KP-1 (Dako), S-100 (Dako), chromogranin (Dako), NSE (neural specific enolase) (Dako), synaptophysin (Dako), *Ulex europaeus* I lectin (Dako), factor VIII (Dako), CD34 (Becton-Dickinson), p53 (Dako), HLA-DR (Coulter, Miami, CA), Leu-7 (Becton-Dickinson) and LCA (leukocyte common antigen) (Becton-Dickinson). Tissue was also taken and fixed in 2.5% glutaraldehyde, and processed according to standard method for electron microscopy.

### *Gross pathology*

The surgically removed tumor was a circumscribed mass with pseudocapsule, measuring 11×10.5×10 cm, weighing 510 g, and revealing a variegated light yellow to tan brown cut surface with extensive yellow discoloration, focal hemorrhage and gelatinous degeneration (Fig. 1B).

### *Microscopic pathology*

The tumor mass consisted of tightly packed spindle-shaped cells surrounding ramified thin-walled endothelium-lined vascular channels with characteristic 'antler' or 'staghorn' configuration (Fig. 2). The neoplastic cells had round to oval to slightly angulated nuclei with clumped chromatin and a moderate amount of cytoplasm with ill-defined cell borders. Mitotic figures were frequently seen with an average of 30 mitoses/10 high power fields, including abnormal ones. Other areas of the tumor revealed extensive necrosis and pseudocapsulation, as well as focal hyaline degeneration. No nerve bundles or trunks were seen in or adjacent to the tumor. Reticulin stain showed that reticular fibers surrounded each individual neoplastic cell, characteristic of hemangiopericytoma (Fig. 3). Neoplastic cells were also present at the inked specimen margins.

### *Immunohistochemistry*

Cell marker study using the immunoperoxidase method revealed that the neoplastic cells were positive for vimentin, factor XIIIa, CD34, HLA-DR, and focally positive for desmin, actin, and myoglobin. These findings were consistent with hemangiopericytoma. Approximately one-third of the tumor cells showed positive nuclear stain by anti-p53 antibody. Tumor cells were negative for S-100, chromogranin, NSE, synaptophysin, Leu-7, precluding its neurogenic origin. There was also no reactivity with antibodies against cytokeratin, *Ulex europaeus* I lectin, factor VIII, KP-1 or α-actin.

### *Electron microscopy*

Ultrastructurally, the tumor cells were closely apposed and spindle-shaped, with large round to oval to slightly angulated nuclei and pale-staining cytoplasm

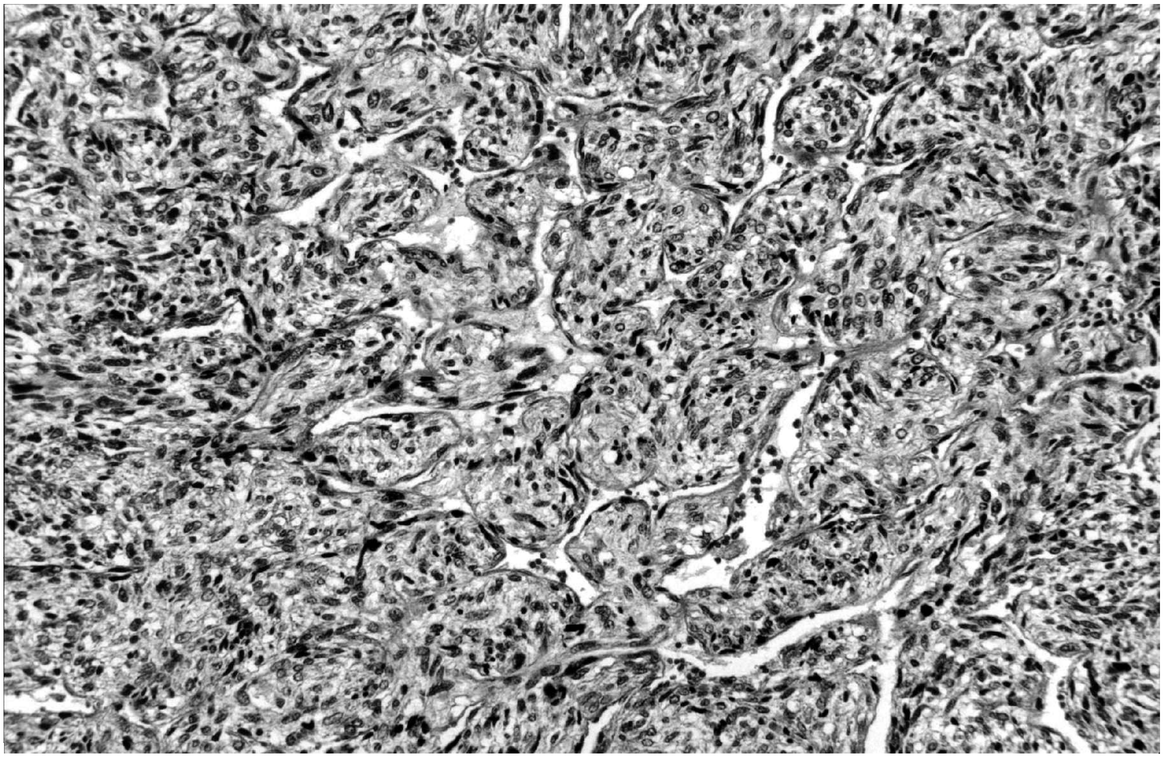

**Fig. 2.** *Histological section of the tumor mass showing tightly packed spindle-like cells surrounding ramifying thin-walled endothelium-lined vascular channels with typical 'antler' or 'staghorn' configuration (H & E, 180×).*

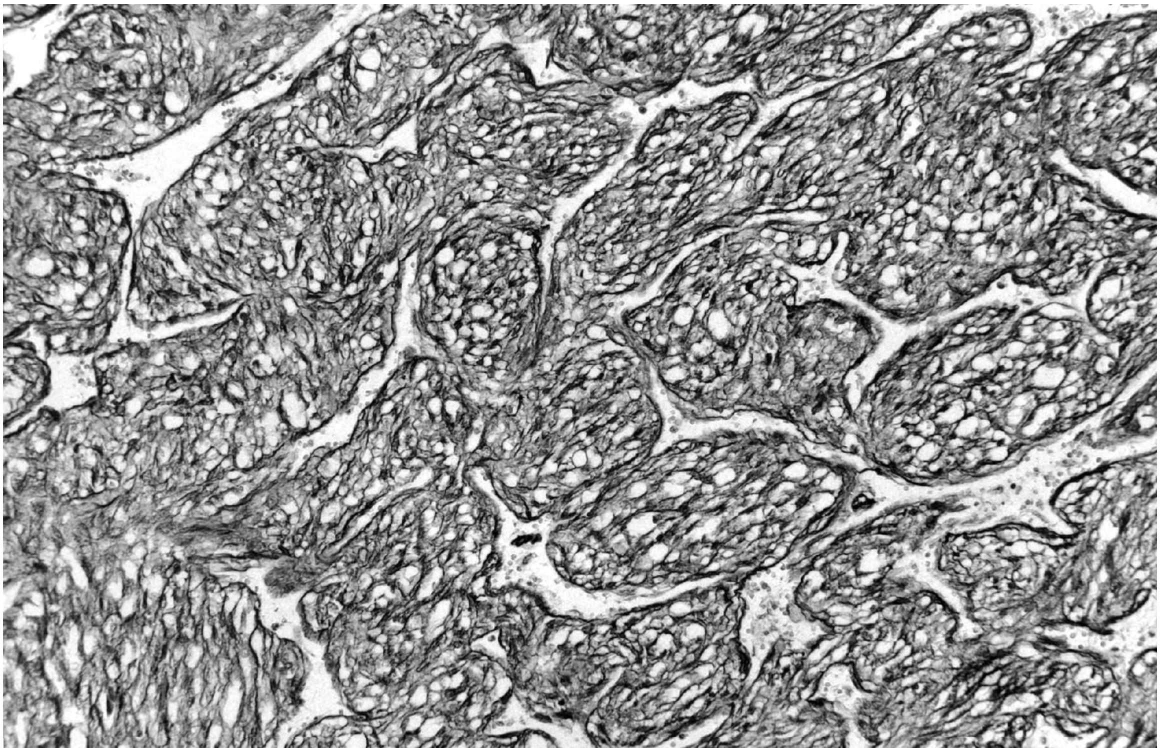

**Fig. 3.** *Reticulin stain showing that reticular fibers surround each individual neoplastic cells (180×).*

containing rough endoplasmic reticulum with few ribosomes, rod-shaped or oval mitochondria, and small bundles of microfilaments. These features are seen in the pericytes. Occasionally, there were myoid cells or myopericytes containing bundles of microfilaments with condensed small fascicles, which were intermediate in appearance between pericytes and smooth muscle cells, and may also explain focal desmin and actin expression. Pinocytotic vesicles were also observed. More characteristically, a distinct occasionally multilayered basal lamina was seen surrounding individual tumor cells. These findings were consistent with hemangiopericytoma. In addition, there was no evidence of dendritic processes characteristic of neurogenic tumors.

*Follow-up*

After discharge the patient was lost to follow-up for 2 months. He returned to the oncology service with a complaint of abdominal pain. CT scan revealed a new left lower peritoneal mass. Five cycles of combined chemotherapy consisting of vincristine, adriamycin, etoposide and ifosiphamide were administered. Follow-up CT scan revealed approximately 60% shrinkage of the mass. The patient is currently undergoing radiation therapy.

### Comment

Neurofibromatosis-1 (NF-1), or von Recklinghausen's disease, is an autosomal dominant disorder affecting 1 in 3000 live births, and is often associated with dermal tumors of neural origin.[1] In addition to cutaneous tumors, patients with NF-1 may have solitary deep tumors including non-neurogenic sarcomas, gastrointestinal ganglioneurofibromatosis, meningiomas, ependymomas and occasionally may show malignant transformation within a neurofibroma.[2–5,7,8] Konishi *et al.* described a case of neurofibromatosis patient with an unusual malignant schwannoma which had a predominant hemangiopericytoma-like arrangement, and also contained foci of rhabdomyosarcomatous and angiosarcomatous differentiation.[7] A hemangiopericytoma and a plexiform neurofibroma present in the same tumor of a patient with NF was also reported by Aduana *et al.*[8] However, the association of solitary hemangiopericytoma with NF is extraordinarily rare. In 1992 Neilly and colleagues reported the first case of a 2.5-cm hemangiopericytoma arising in the ileum in a patient with von Recklinghausen's disease.[5] We describe here another case of malignant hemangiopericytoma in a 27-year-old man with NF-1.

Since the morphology of hemangiopericytoma-like pattern or arrangement can be seen focally in a variety of tumors including benign and malignant fibrous histiocytoma, synovial sarcoma, mesenchymal chondrosarcoma, nerve sheath tumors, cellular hemangioma, glomus tumor, and leiomyosarcoma, differentiation of hemangiopericytomas from these tumors is necessary, particularly from the first four entities.[1,6] A combination of histological, immunohistochemical, and ultrastructural studies on multiple sections will help to differentiate hemangiopericytoma from those tumors mimicking its pattern. If a tumor shows a consistent hemangiopericytoma (uniform cellularity and vascular) pattern throughout the entire tumor, with the dense reticulin meshwork surrounding the individual tumor cells, and is negative for muscle, nerve sheath, and epithelial markers, but positive for pericyte markers, such as CD34 and factor XIIIa, etc., the diagnosis of hemangiopericytoma can be established in most cases.[6] As in the present case, the tumor revealed not only a uniform hemangiopericytoma pattern throughout the entire mass, but a consistent immunohistochemical and ultrastructural characterization of hemangiopericytoma as well. There was no evidence of any neurogenic differentiation.

The NF-1 gene, belonging to the family of tumor suppressor genes, has been mapped to chromosome 17q11.2, encoding a protein, neurofibromin, which down-regulates the function of the p21 *ras* oncoprotein, and NF-1 is associated with deletions, insertions or mutation in the NF-1 gene region.[9] Various abnormalities on chromosome 17 but outside the NF-1 gene region, possibly involving the p53, have been demonstrated.[10] In the present case we also demonstrated p53 positivity in about one-third of the tumor cells. The exact mechnism(s) of the malignant transformation or tumor progression or the tumorigenesis of non-neurogenic tumors in neurofibromatosis 1 is not yet fully understood.

In summary, we described a 27-year-old male with NF-1 who developed a malignant hemangiopericytoma in the pelvis. The case reported here serves as a reminder, especially for surgical pathologists, that although extremely rare hemangiopericytoma may occur in patients with NF and it should be included in the differential diagnosis along with other non-neurogenic sarcomas, particularly when a histology of hemangiopericyte-like pattern is encountered in solitary tumors in these patients.

### References

1. Riccardi V. Von Recklinghausen's neurofibromatosis. *N Engl J Med* 1981; 305:1617–26.
2. Harkin JC, Reed RJ. Tumor of the peripheral nerve system. In: Firminger HI, ed. *Atlas of tumor pathology*, Second Series, Fascicle 3. Washington, DC: Armed Forces Institute of Pathology 1968; 107–36.
3. Woodruff JM, Chernik NL, Smith MC, *et al.* Peripheral nerve tumors with rhabdomyosarcomatous differentiation (malignant 'triton' tumors). *Cancer* 1973; 32:426–39.
4. Macaulay RAA. Neurofibrosarcoma of the radial nerve in von Recklinghausen's disease with metastatic angiosarcoma. *J Neurol Neurosurg Psychiatry* 1978; 41:474–8.
5. Neilly PJ, Cooper GG, O'Hara MD, McGrady BJ. Ileal haemangiopericytoma and von Recklinghausen's disease. *Br J Clin Pract* 1992; 46:212–3.
6. Enzinger FM, Smith BH. Hemangiopericytoma: an analysis of 106 cases. *Hum Pathol* 1976; 7:61–82.
7. Konishi N, Hiasa Y, Shimoyama T, *et al.* Malignant 'triton' tumor with metastatic hemangiopericytoma in a patient associated with von Recklinghausen's disease. *Acta Pathol Japon* 1986; 36:459–69.
8. Aduana V, Mohammadi H, Vaiana J, Ghosh L. Hemangiopericytoma arising in a solitary plexiform neurofibroma: report of a case and review of the literature. *J Oral Maxillofac Surg* 1988; 46:1106–9.
9. Gutmann DH, Collins FS. The neurofibromatosis type 1 gene and its protein product, neurofibromin. *Neuron* 1993; 10:335–43.
10. Menon AG, Anderson KM, Riccardi VM, *et al.* Chromosome 17p deletions and p53 gene mutations associated with the formation of malignant neurofibrosarcoma in von Recklinghausen's neurofibromatosis. *Proc Natl Acad Sci USA* 1990; 87:5435–9.
